# Tuning the trabecular orientation of Voronoi-based scaffold to optimize the micro-environment for bone healing

**DOI:** 10.1007/s10237-025-01953-8

**Published:** 2025-04-24

**Authors:** Luca D’Andrea, Giorgio Goretti, Gianni Magrini, Pasquale Vena

**Affiliations:** 1 Department of Chemistry, Materials and Chemical Engineering “Giulio Natta”, Laboratory of Biological Structure Mechanics (LaBS), Politecnico di Milano, Piazza Leonardo da Vinci 32, 20133 Milan, Italy; 2 Faculty of Medicine and Health Technology, Laboratory of Biomaterials and Tissue Engineering, Tampere University, Korkeakoulunkatu 3, 33720 Tampere, Finland; 3 Lithoz GmbH, Mollardgasse 85A/2/64-69, 1060 Vienna, Austria; 4 MedApp SA, Al. Juliusza Słowackiego 6/10, 30-037 Krakow, Poland

**Keywords:** Voronoi, Mechanical properties, Tissue engineering, Finite element, Bone scaffold

## Abstract

Voronoi tessellation is a powerful technique for designing random structures for bone tissue engineering applications. In this study, an innovative algorithm for scaffold design that controls trabecular orientation while maintaining an overall random architecture is presented. Morphological analyses and numerical models were employed to comprehensively characterize the scaffolds. The results indicate that the effective stiffness and permeability of the scaffolds are directly influenced by the trabecular orientation. In contrast, other parameters, such as porosity, trabecular thickness, trabecular spacing, and curvatures, can be kept constant with respect to the trabecular orientation. These findings, in conjunction with mechano-biological considerations, provide a robust design workflow to optimize the micro-environment for bone growth. This framework offers a valuable tool for selecting the most suitable scaffold architecture according to the specific external loads, thereby enhancing the efficacy and reliability of bone scaffolds in clinical applications. Through this approach, the aim is to improve the precision and outcomes of bone tissue engineering, contributing to the development of advanced therapeutic solutions for bone repair and regeneration.

## Introduction

The need for bone tissue implants is increasing worldwide due to the aging of global population (Ravazzano et al. 2024). Bone grafts or artificial scaffolds can be used to treat fractures and replace tissue in bone abnormalities (Zeng et al. 2018). A suitable scaffold design aims to simultaneously optimize its mechanical properties and biological response (Breuls et al. 2008) in a multidisciplinary context. Microstructured architectures can be classified in regular (periodic) and random (stochastic) lattices (Chen et al. 2020; Nemes-Károly and Szebényi 2023; Foroughi et al. 2024). Even if trabecular bone seems to be randomly oriented, from the pioneering work of Culmann and Von Meyer it is evident that bone tissue is arranged according to some preferential directions, or stress lines (Cowin 2001). In the search for the optimal geometry for scaffold design, too often attention has been focused on replicating the architecture of trabecular bone. It is worth mentioning that trabecular bone is optimized according to its material constituents (i.e., composite of collagen and hydroxyapatite) and hierarchical structure (Rho et al. 1998). Due to the impossibility of reproducing such complex material in bone scaffolds, it is more convenient, instead, to design architectural features that, depending on the properties of the constituent material of the device, can replicate the overall properties of trabecular bone. Voronoi-based architectures can be used as bone scaffolds. Different studies focusing on the design of Voronoi structures as bone scaffolds have been reported, using a traditional formulation of the Voronoi tessellation (Wang et al. 2022), taking inspiration from the trabecular bone (Gómez et al. 2016), or using deep learning (Zheng et al. 2023). Other studies tailored the mechanical anisotropy by stretching the architecture along one direction (Vaiani et al. 2023) or introducing a different number of seeds (Zhou et al. 2023; Rezapourian and Hussainova 2023). However, in most cases, the anisotropy is weakly controlled and pores interconnection is lost. More generally, the scaffold design is a multi-objective optimization process, where many dependent and independent variables have to be determined. Josephson and Morgan (2024) explored many triply periodic minimal surfaces scaffolds generating a suitable micro-environment for bone cell growth, like curvature, octahedral shear strain, fluid shear strain and stiffness. Unlike triply periodic minimal surfaces that are strictly dependent on the analytical definition, stochastic architectures have a more flexible design allowing for an independent tuning of different features or parameters. Portela et al. (2020) applied an anisotropic energy-based criterium to generate thin walled bicontinuous structures providing a mechanical anisotropy. However, the labyrinthine nature of the structure may limit the permeability and the diffusion of nutrients needed for cell proliferation. Chao et al. (2021) combined computational and experimental analyses on Voronoi scaffolds, evaluating how the geometric and mechanical properties affect the cellular growth. Although the trabecular thickness, trabecular spacing and porosity were taken into account in the design of the structures, no reference to the trabecular orientation was reported.

In this work, a novel methodology to generate micro-architected scaffolds with prescribed preferential directions of the trabeculae and an independent control on the trabecular thickness and the overall porosity based on the Voronoi tessellation is proposed. The anisotropic elastic characteristics of the produced scaffolds were evaluated using computational models, and their capacity to stimulate tissue growth was evaluated by determining superficial octahedral strain and overall fluid permeability.

## Materials and methods

### Design algorithm

The design of the scaffold started from the definition of its volume domain, arbitrarily identified as a cube with 500 units of length per edge through a MATLAB script. This value was chosen to have a good compromise between the macroscopic volume of the scaffold and the number of voxels per trabecula (providing a good discretization across the thickness). Inside this volume, 300 points, referred to as seeds, were randomly placed such that the Euclidean mutual distance was higher than 25 units of length. The latter was introduced to avoid the clustering of the seeds. A density of seed can thereby defined as the ratio of the number of seeds (300) and the volume ($500^3$), in this case equal to $2.4\cdot 10^{-6}$; few seeds provide a higher degree of randomness but lower connectivity, whereas, more seeds provide higher connectivity but lower randomness, being them more regularly distributed in the volume. Thus, the Voronoi tessellation was built subdividing the space into polyhedra, where each polyhedron encompasses one single seed and all the points of the space that are closer to that seed than to any other

1 $$\begin{aligned} V(p_i) = \{ p \,|\, d(p, p_i) \le d(p, p_j),\, j \ne i,\, j = 1, \ldots , n \} \end{aligned}$$

where the Voronoi cell $V(p_i)$, associated with the seed $p_i$, is the set of all points whose distance from $p_i$ is lower or equal than their distance from any other seed $p_j$, where *j* is any index different from *i*. In this work, the edges of the Voronoi polyhedra were used to generate the rod-like trabeculae of the scaffold.

The *z* axis is here used as reference to identify anisotropic response and trabecular orientations. The orientation ($\vartheta _z$) of every edge with respect to the z-axis was computed as

2 $$\begin{aligned} \vartheta _z = cos^{-1} \left( \frac{v_z}{|{\textbf {v}}|} \right) \end{aligned}$$

where $v_z$ is the *z* Cartesian component of the vector ${\textbf {v}}$, and $|\cdot |$ the modulus operator. A total number of 5000 edges was set as the target number of the final Voronoi tessellation. In order to provide a preferential orientation of the edges, the angular distance ($\delta$) was defined as

3 $$\begin{aligned} \delta = |\vartheta _z - \beta _z| \end{aligned}$$

where the angle $\beta _z$ represents the target angle. The $70\%$ of the desired edges were selected by only considering their distance from $\beta _z$, and keeping the ones with the lower $\delta$. The remaining $30\%$ were selected through a random-based probability selection algorithm, by assigning to each edge a probability value (${\mathcal {P}}$) as  

4 $$\begin{aligned} {\mathcal {P}} = \frac{\delta _{max} - \delta }{\delta _{max}} \end{aligned}$$

where $\delta _{max}$ was the maximum distance from the target angle $\beta _z$ found among all the edges. The probability, defined in Eq. 4, approached 1 for edges aligned with $\beta _z$ and 0 for those which were far apart from it. Then, the probability assigned to every edge was compared with a random value (between 0 and 1), generated for every comparison; the edges with a probability higher than the random values are saved, until reaching the desired number of edges (i.e., 5000). This method allowed to select mainly the trabeculae with an orientation closer to the target angle without excluding completely all the others, thus enhancing the anisotropy of the model maintaining the interconnection in the structure. After a parametric analysis of the final connectivity, the percentage of randomly chosen trabeculae was fixed at 30% (data not shown). Microstructures with unconnected sections are produced by a percentage below 30%; on the other hand, a higher percentage will result in less control over the final structure’s anisotropic properties.

In case of a target isotropic structure, a different procedure was followed to avoid any preferential direction in the trabecular orientation. All the angles of the edges $\vartheta _z$ were subdivided into 9 intervals, from $0^{\circ }$ to $90^{\circ }$, evenly spaced of $10^{\circ }$. Then, the edges composing the scaffolds were selected one by one in a random way, from the first group to the last. This procedure was repeated iteratively until the total desired number of trabeculae (i.e., 5000) was addressed.

After the selection process, the edges disconnected from the main structure were discarded. Finally, the edges were thickened by defining a 3D matrix with pixel size equal to the unit of length; to this purpose, the pixels whose centroid is within half of a prescribed trabecular thickness distance from the segment is set to 1, the remaining pixels are set to 0. The trabecular structure is transformed into a stack of binary images.

The 3D binary images were converted to an stl file to generate the closed surface of the scaffold. A regularization and smoothing of the sharp curvatures were achieved by minimization of the surface energy with a constraint on the enclosed volume. To this purpose the software Surface Evolver was used (Brakke 1992). 50 consecutive evolutions were performed minimizing the surface energy, imposing a target volume of the solid phase of the scaffold. Through this procedure, and using 3 different target volumes, corresponding to 3 porosities ($\phi$), namely 60, 75 and 90%, the final scaffolds were generated (starting from the same seed distribution and trabecular selection).

Finally, the results were converted in tiff 3D binary stacks by intersecting the stl with a 3D matrix of 500 pixel per edge (Patil and Ravi 2005). The external layers of the structure, consisting of 15 pixel per face, were removed to eliminate the trabeculae lying on the faces of the bounding box, since they are typically highly oriented along the Cartesian planes.

### Morphological analyses

The number of edges converging in one node was evaluated for each preferential orientation of the scaffolds and the angle of edges converging in the same node was evaluated (inter-trabecular angle) (Reznikov et al. 2016). When more than two edges converged in the same node, each combination of edge pairs was considered.

The morphological analyses were performed by Fiji^©^, with the purpose to compute the trabecular thickness (*Tb*.*Th*) and the trabecular spacing (*Tb*.*Sp*) of the scaffolds (Dougherty and Kunzelmann 2007). Furthermore, the degree of anisotropy (*DA*) was computed as reported in (Odgaard 1997), namely

5 $$\begin{aligned} DA = 1-\frac{\lambda _{min}}{\lambda _{max}} \end{aligned}$$

where $\lambda _{min}$ and $\lambda _{max}$ are the minimum and maximum eigenvalues of the fitted ellipsoid obtained through the Mean Intercept Length algorithm, respectively.

The Gaussian curvature of the scaffolds, defined as the product between the two principal curvatures ($\chi _{min}$ and $\chi _{max}$) was computed in MeshLab.

The pixel size was set to $15\; \mu m$ such that both the *Tb*.*Th* and the *Tb*.*Sp* are suitable for additive manufacturing in metal (Pesode and Barve 2022), ceramic (Dadkhah et al. 2023) or polymeric (Pal et al. 2021) material and cellular proliferation (Xia and Luo 2022), respectively.

### Mechanical analyses

The scaffolds were subjected to a homogenization process through the finite element solver ParOSol (Flaig and Arbenz 2011), to compute the stiffness matrix ${\varvec{C}}$ and consequently the effective mechanical parameters [28]. This process provides the normalized stiffness along any spatial orientation in the 3D space (${\bar{E}}({\varvec{n}})$):

6 $$\begin{aligned} {\bar{E}}({\varvec{n}})=\frac{1}{n_in_jn_kn_lD_{ijkl}} \end{aligned}$$

in which the $D_{ijkl}$ is the fourth order compliance tensor, i.e., the inverse of the stiffness tensor $C_{ijkl}$. The macroscopic elastic modulus of a scaffold manufactured with a specific material will be $E({\varvec{n}}) = E_0 {\bar{E}}({\varvec{n}})$, where $E_0$ is the intrinsic stiffness of the selected material.

As done for the geometric anisotropy *DA*, the index of mechanical anisotropy ($A^Z$) was computed as

7 $$\begin{aligned} A^Z = 1-\frac{\bar{E}_{min}}{\bar{E}_{max}};\;\;\; E_{max}=\max _{{\varvec{n}}} ({\bar{E}}({\varvec{n}})); \;\;\; E_{min}=\min _{{\varvec{n}}} ({\bar{E}}({\varvec{n}})) \end{aligned}$$

In order to take into account the anisotropy given by the shear moduli, the tensorial anisotropy index ($A^T$) was computed starting from the components of the stiffness matrix ${\varvec{C}}$ as

8 $$\begin{aligned} A^T = \frac{2(C_{44}+C_{55}+C_{66})}{(C_{11}+C_{22}+C_{33})-(C_{12}+C_{13}+C_{23})}+\sum _{n=1}^{3} \alpha (C_{G_i}) \end{aligned}$$

where $C_{ij}$ are the components of the stiffness matrix ${\varvec{C}}$ and $\alpha (C_{G_i})$ is the coefficient of variation for every stiffness group accounting for directional stiffness differences (Ranganathan and Ostoja-Starzewski 2008).

In order to compare the stiffness along the z-direction in groups of scaffolds exhibiting the same porosity, the normalized effective stiffness on the z-direction ($\langle E_z \rangle$) was computed as

9 $$\begin{aligned} \langle E_z \rangle = \frac{{\bar{E}}_z^{\beta _z}}{{\bar{E}}_z^{ISO}} \end{aligned}$$

where ${\bar{E}}_z^{\beta _z}$ is the effective elastic modulus along the z-direction for the selected scaffold with target orientation $\beta _z$ and ${\bar{E}}_z^{ISO}$ the effective elastic modulus of the scaffold without preferential direction (here named as isotropic).

### Properties related to tissue growth: fluid permeability and superficial strain

In this section, two properties that are important in the eventual capability to support tissue growth in the scaffold are proposed. In particular, the macroscopic fluid permeability, which allows for nutrient transport, and superficial strain that may promote cell response. In order to determine macroscopic permeability of the scaffold, it is assumed that the porosity of the microstructure is filled with a micro-porous substance like a collagen gel (Mohee et al. 2019; Dewey et al. 2023) or a granulation tissue (Ghimire et al. 2021) which has a small-scale permeability $\kappa _0$ that mostly depends on its intrinsic microporosity (O’Brien et al. 2007). A further assumption is that the fluid flow through the microporosity of the collagen gel or granulation tissue is described through the Darcy law:

10 $$\begin{aligned} \textbf{q}_{\mu }=\kappa _{0} \nabla p \end{aligned}$$

in which $\textbf{q}_{\mu }$ is a vector the entries of which are the local volume fluid flux along the three Cartesian directions; while *p* is the fluid pore pressure.

By combining the Darcy law (equation 10) with the mass conservation law and assuming: i) a relatively rigid solid scaffold, ii) fluid incompressibility and iii) homogeneous distribution of the intrinsic small-scale permeability, one has:

11 $$\begin{aligned} \kappa _0 \nabla ^2 p =0 \end{aligned}$$

and related boundary conditions, i.e., impervious walls at the interface with the solid scaffold.

Let’s consider a cubic macroscopic domain with edge length *L* such that $L>>d_{ps}$ in which $d_{ps}$ is the typical pore size of the scaffold. Consider also this cubic domain as a domain made of an equivalent homogeneous material characterized by a macroscopic permeability ${\varvec{K}}$ which is in general a tensorial quantity (here represented as a matrix with three rows and three columns) and a macroscopic fluid flux along the three Cartesian directions $\textbf{q}$. For this cubic domain the macroscopic Darcy equation states:

12 $$\begin{aligned} \textbf{q}=\textbf{K} \nabla p \end{aligned}$$

If now a cubic Representative Volume Element (RVE) of a scaffold is considered, in which the pores of the microstructure are explicitly represented, one can define the relationship between the local fluid flux $\textbf{q}_\mu$ and the macroscopic cubic flux $\textbf{q}$ as (Bear 1972; Renard et al. 2001):

13 $$\begin{aligned} \textbf{q}= & \frac{1}{V_{RVE}} \int _{V_{RVE}} \textbf{q}_{\mu } dV \end{aligned}$$

14 $$\begin{aligned} \textbf{K} \nabla p= & \frac{1}{V_{RVE}} \int _{V_{RVE}} \textbf{q}_{\mu } dV \end{aligned}$$

The components of the tensor ${\varvec{K}}$ can be obtained by running three different finite element analyses in which the RVE is subjected to a unit pressure gradient in each spatial direction.

Let’s consider the first FEM:

15 $$\begin{aligned} ^1\textbf{q}=\left[ \begin{array}{c} 1\\ 0\\ 0 \end{array} \right] \end{aligned}$$

This macroscopic condition can be achieved by setting the boundary conditions on all faces $\Gamma$ of the RVE as:

16 $$\begin{aligned} p|_{\Gamma }=x \end{aligned}$$

The finite element analysis run using the boundary conditions 16 will provide a local (microscopic) distribution of fluid volume flux $^1\textbf{q}_\mu$. By combining 13 and 14 one gets:

17 $$\begin{aligned} K_{11}&=-\frac{1}{V_{RVE}} \int _{V_{RVE}} {}^1q^1_{\mu } dV; \end{aligned}$$

18 $$\begin{aligned} K_{12}&=-\frac{1}{V_{RVE}} \int _{V_{RVE}} {}^1q^2_{\mu } dV; \end{aligned}$$

19 $$\begin{aligned} K_{13}&=-\frac{1}{V_{RVE}} \int _{V_{RVE}} {}^1q^3_{\mu } dV; \end{aligned}$$

in which $^1q^i_{\mu}$ is the $i-th$ Cartesian component of the vector $^1\textbf{q}_{\mu }$. By running the FEM simulations on the RVE by using the following boundary conditions:

20 $$\begin{aligned} p|_{\Gamma }=y \end{aligned}$$

and

21 $$\begin{aligned} p|_{\Gamma }=z \end{aligned}$$

all entries of the ${\varvec{K}}$ matrix can be obtained.

In order to determine the macroscopic permeability along any arbitrary spatial direction $\textbf{n}$ the following relationship can be used:

22 $$\begin{aligned} k_n=\kappa _0\textbf{n}^T\textbf{K} \textbf{n} \end{aligned}$$

As done for the geometrical and mechanical anisotropy *DA* and $A^Z$, an anisotropy index for the permeability ($A^\kappa$) was computed as

23 $$\begin{aligned} A^\kappa = 1- \frac{\kappa _{min}}{\kappa _{max}} \end{aligned}$$

where $\kappa _{min}$ and $\kappa _{max}$ are the minimum and maximum eigenvalues of the matrix ${\varvec{K}}$, respectively. This index thus provides a scalar measure of permeability anisotropy, enabling better characterization of the material’s flow properties across various spatial orientations.

Observing the phenomenological law described in Huiskes et al. (1997), there is a direct dependency between the mechano-biologic stimulus (*S*) and the octahedral shear strain ($\gamma _{oct}$) of the substrate on which cells are located, as reported in the following equation

24 $$\begin{aligned} S = \frac{\gamma _{oct}}{a}+\frac{v}{b} \end{aligned}$$

where *a* and *b* are constants and *v* is the velocity of a fluid flowing in the structure. The parameter *S* represents a tool to predict the bone growth under an external load and a fluid flowing in the porosity. Thereby, uniaxial compression and strip uniaxial compression were performed and the $\gamma _{oct}$ was computed on the external first layer of voxels of the structure (i.e., on the surface of the solid scaffold), as

25 $$\begin{aligned} \gamma _{oct} = \frac{2}{3} \sqrt{(\varepsilon _{1}-\varepsilon _{2})^{2}+(\varepsilon _{2}-\varepsilon _{3})^{2}+(\varepsilon _{3}-\varepsilon _{1})^{2}} \end{aligned}$$

where $\varepsilon _{1}$, $\varepsilon _{2}$ and $\varepsilon _{3}$ are the principal strains.

## Results

### Morphological analyses

**Fig. 1:**
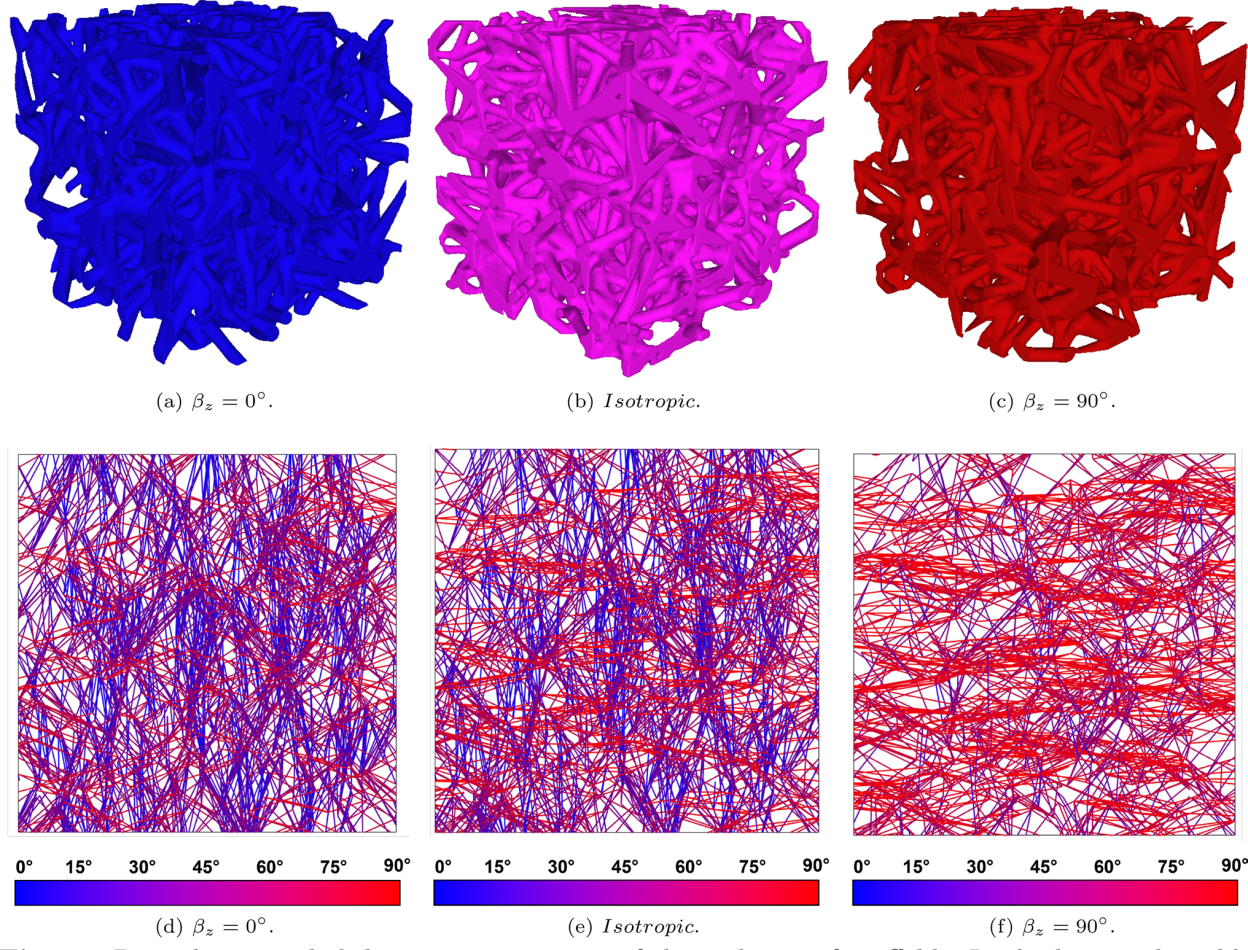
3D rendering and skeleton representation of three classes of scaffolds. In the bottom line, blue trabeculae represent vertical trabeculae, red trabeculae represent horizontal trabeculae. The color combination of blue and red represent diagonal trabeculae

A visual representation of the angle between the edges after the selection algorithm and the vertical direction (z-direction) is reported in Fig. 1, red color refers to trabeculae mostly perpendicular to the vertical axis, while blue color refers to trabeculae mostly oriented along the vertical direction. The figure shows how the selection of the trabecular orientation while keeping structure connectivity achieved the desired result.

Figure 1 shows that for the isotropic case (panel (b)) there is not a clear prevailing color (panel (e)). Concerning the preferential direction $\beta _z=0^{\circ }$ (panel (a)) and $\beta _z=90^{\circ }$ (panel (c)) there is a prevailing presence of blue (panel (d)) and red (panel (f)), respectively.

**Fig. 2:**
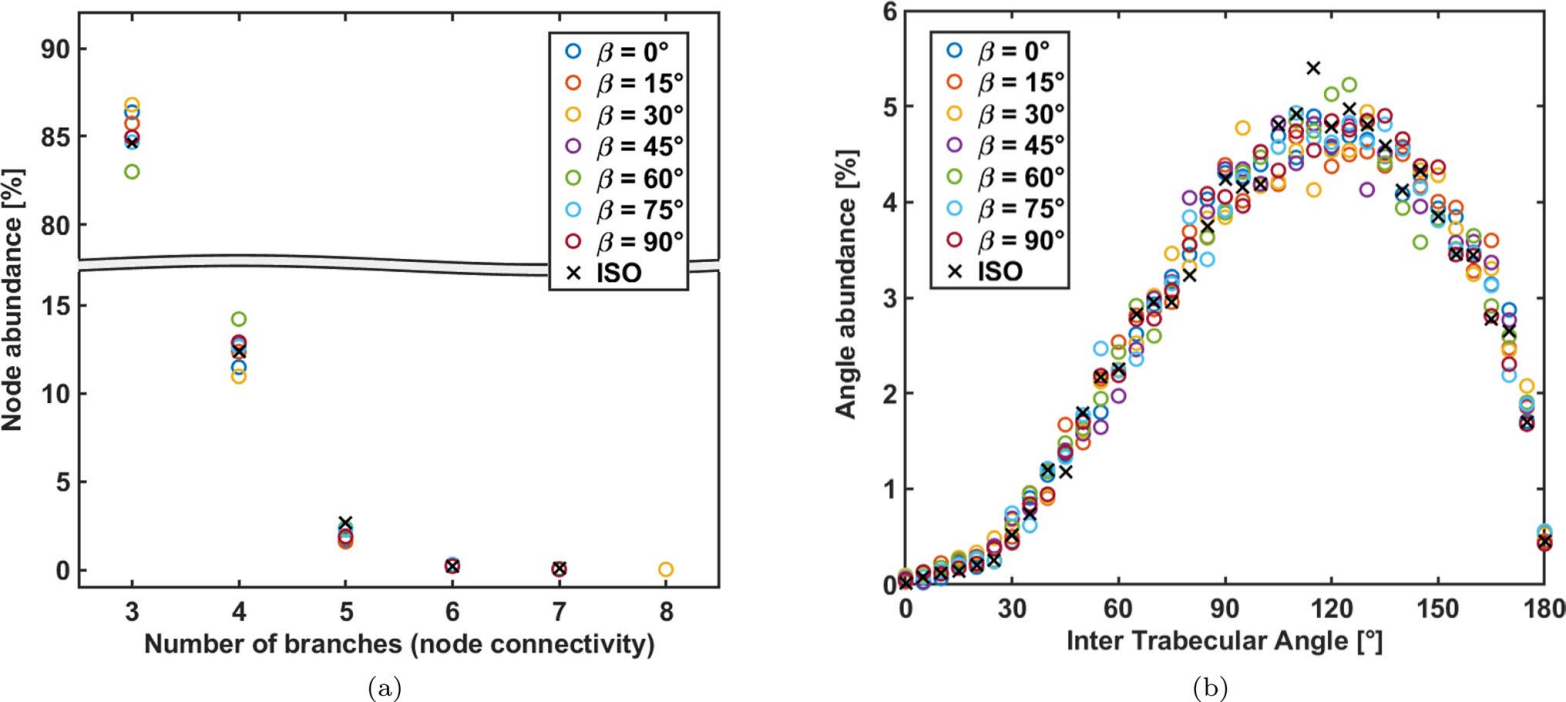
Panel **a**: number of branches converging in one node; panel **b**: inter-trabecular angle among each node

The number of trabeculae converging in each node is reported in Fig. 2a and the inter-trabecular angle in Fig. 2b.

All configurations exhibit a number of trabeculae converging in one node that ranges from 3 to 6, with a similar node abundance trend. The highest incidence is represented by 3 trabeculae converging in one node, and it decreases as the number of branches increases. Few orientations exhibit a number of branches up to 8, but they represent only $1\%$ of the total occurrences. The inter-trabecular angle distribution is similar among all the preferential orientations $\beta _z$, exhibiting a peak when the inter-trabecular angle is around $120^{\circ }$ which is consistent with the highest incidence of 3 trabeculae converging in the nodes (Reznikov et al. 2016).

**Fig. 3:**
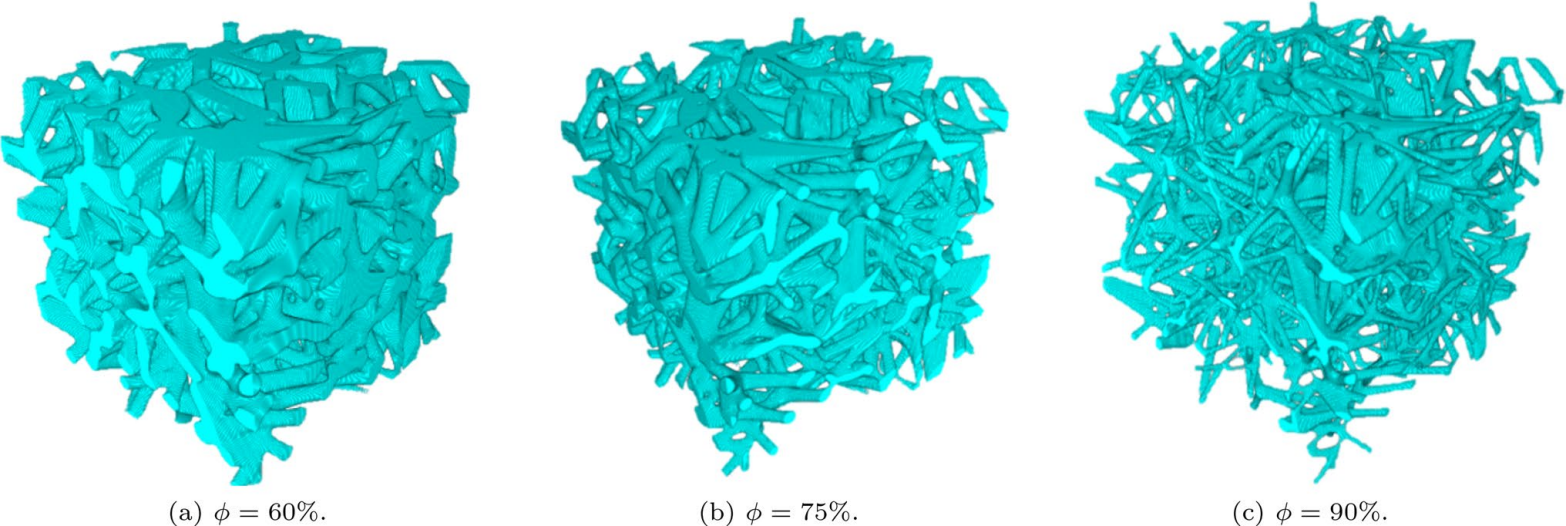
Rendering of the isotropic scaffolds belonging to the three classes of porosity

As imposed on the constrained minimization of the surface energy, the 3 classes of scaffolds exhibit a porosity of 60, 75 and 90%. The 3D rendering of the three scaffolds belonging to the aforementioned porosities is reported in Fig. 3.

**Fig. 4:**
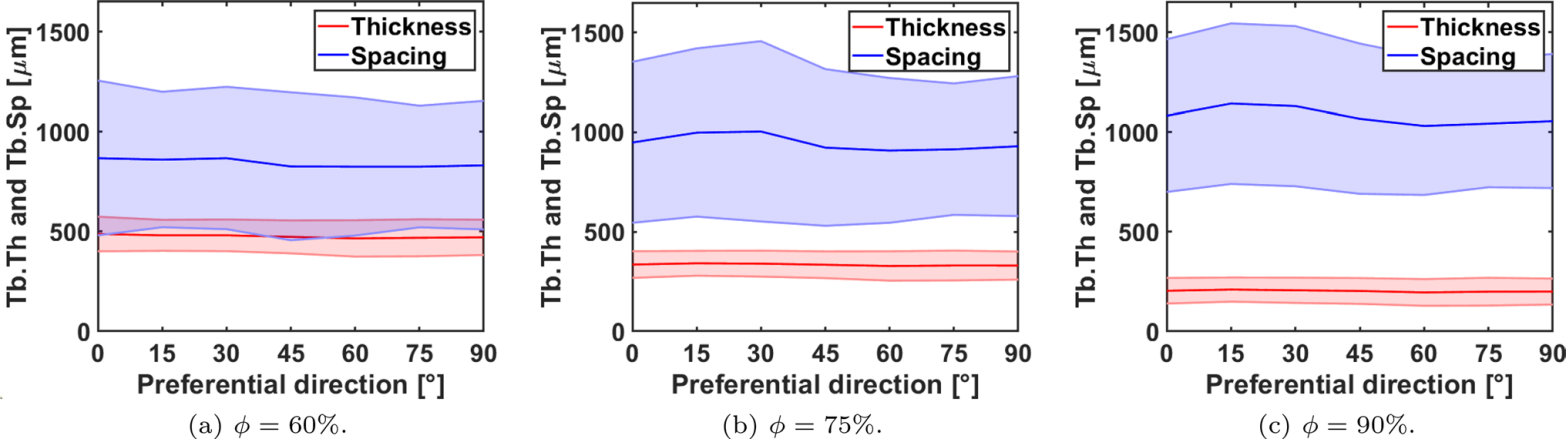
Trabecular thickness and trabecular spacing as a function of the preferential direction of the trabeculae

The mean trabecular thickness is roughly constant for each preferential orientation (Fig. 4), meaning that the minimization of the surface energy in Surface Evolver acts uniformly on the whole volume.

As expected the mean value of the *Tb*.*Sp* slightly increases with the porosity, while the *Tb*.*Th* decreases. In particular, the *Tb*.*Th* and the *Tb*.*Sp* for the three classes of porositiy, from the less porous to the most porous, are $Tb.Th = 450\; \mu m \pm 90\; \mu m$ and $Tb.Sp = 825\; \mu m \pm 360\; \mu m$, $Tb.Th = 330\; \mu m \pm 75\; \mu m$ and $Tb.Sp = 900\; \mu m \pm 375\; \mu m$, $Tb.Th = 195\; \mu m \pm 60\; \mu m$ and $Tb.Sp = 1020\; \mu m \pm 360\; \mu m$, respectively.

**Fig. 5:**
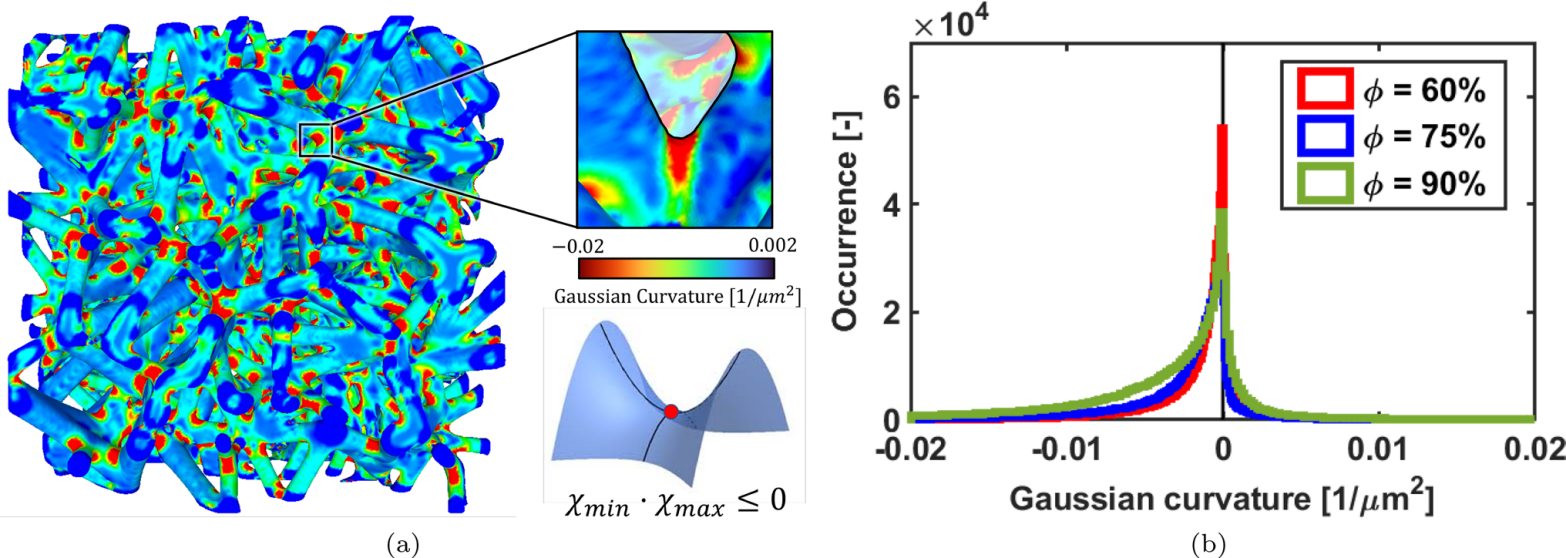
Panel **a**: rendering of the Gaussian curvature of an isotropic scaffold ($\phi = 75\%$); panel **b**: histogram of the Gaussian curvature for the three isotropic scaffolds

The visual representation of the Gaussian curvature is reported in Fig. 5. As a representative example the isotropic scaffold with $\phi =75\%$ is reported (panel (a)).

High negative values are present in the interconnections of the trabeculae (saddle areas), whereas values close to zero represent the external surface of trabeculae far from the nodes (panel (b)). The shape of the three histograms in Fig. 5b is similar for three scaffolds, with a slight presence of more occurrence in the negative Gaussian curvature for $\phi = 90\%$; this is justified by the fact that thinner structures exhibit less surface in the central areas of the trabeculae than the interconnecting points. The positive part of the Gaussian curvature is generated by pits (both negative principal curvatures) or peak (both positive principal curvatures) however, both cases are negligible in the structure, which is consistent with a beam-like architecture.

### Elastic properties

**Fig. 6:**
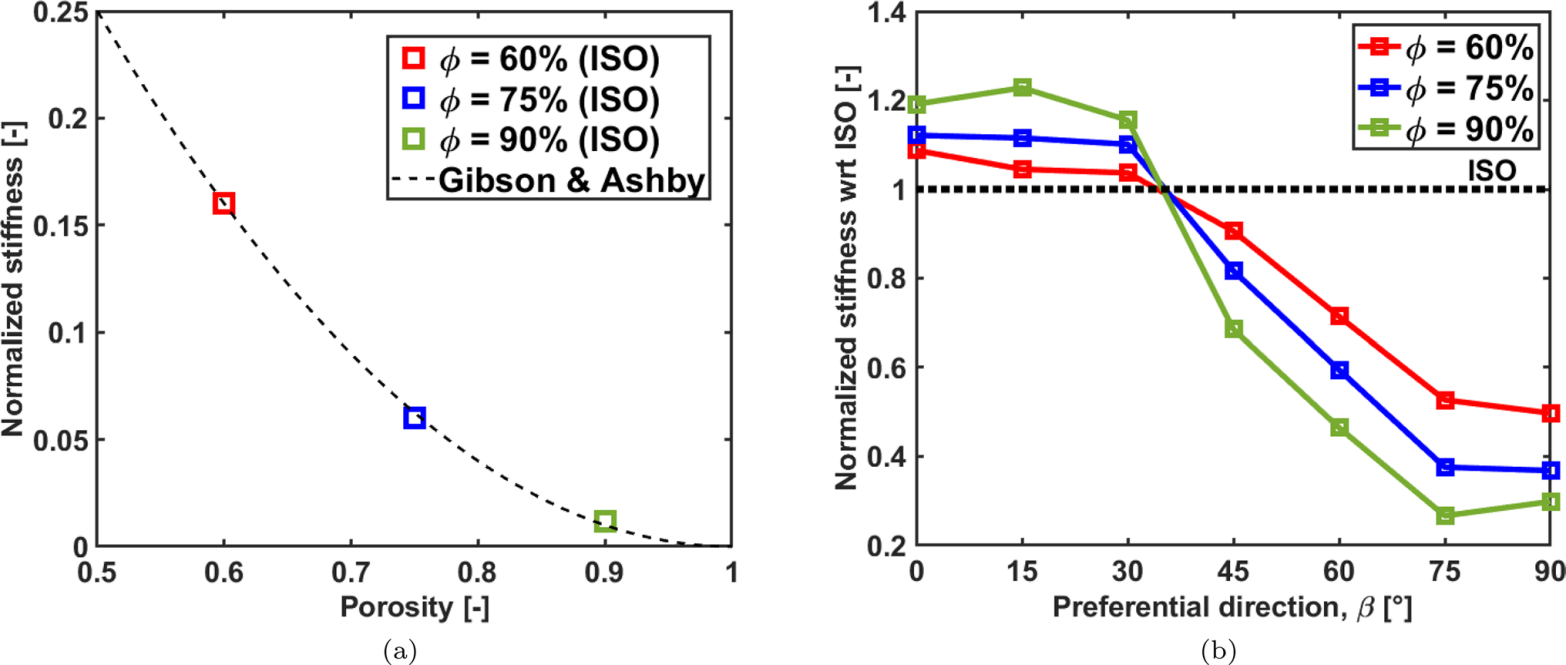
Panel **a**: comparison of the value of stiffness of the three isotropic scaffolds with Gibson and Ashby prediction (Gibson and Ashby, 1982); panel **b**: comparison of the effective stiffness of the isotropic scaffolds with respect to the stiffness of the scaffolds exhibiting a preferential orientation of the trabeculae

The normalized stiffness ${\bar{E}}_z$ for the isotropic configuration for three selected porosities is reported in Fig. 6a. The three values nicely overlap with the prediction provided by Gibson and Ashby for bending-dominated structures(${\bar{E}}=E/E_0=C(1-\phi )^2$ with $C=1$) (Gibson and Ashby, 1982). The stiffness along the vertical direction for the scaffolds with preferential orientation of the trabeculae is shown in 6(b) in a normalized form as ${\bar{E}}^{\beta _z}_z /{\bar{E}}^{ISO}_z$, being ${\bar{E}}^{ISO}_z$ the stiffness along the vertical direction for the scaffolds with no preferential orientations.

As expected, the most porous scaffolds show the highest impact on the mechanical anisotropy, with an increase of the vertical stiffness with respect to the reference isotropic scaffold of approximately 20% for the microstructures with $\beta _z=0^{\circ },15^{\circ },30^{\circ }$ while the most compliant scaffold with 90% of porosity is achieved with preferential orientations more perpendicular to the vertical orientation $\beta _z=75^{\circ },90^{\circ }$. A similar trend is found for $\phi =75\%$ and $\phi =60\%$, with a reduction of the stiffness variation with respect to the isotropic case, that decreases when the porosity decreases.

### Anisotropy

**Fig. 7:**
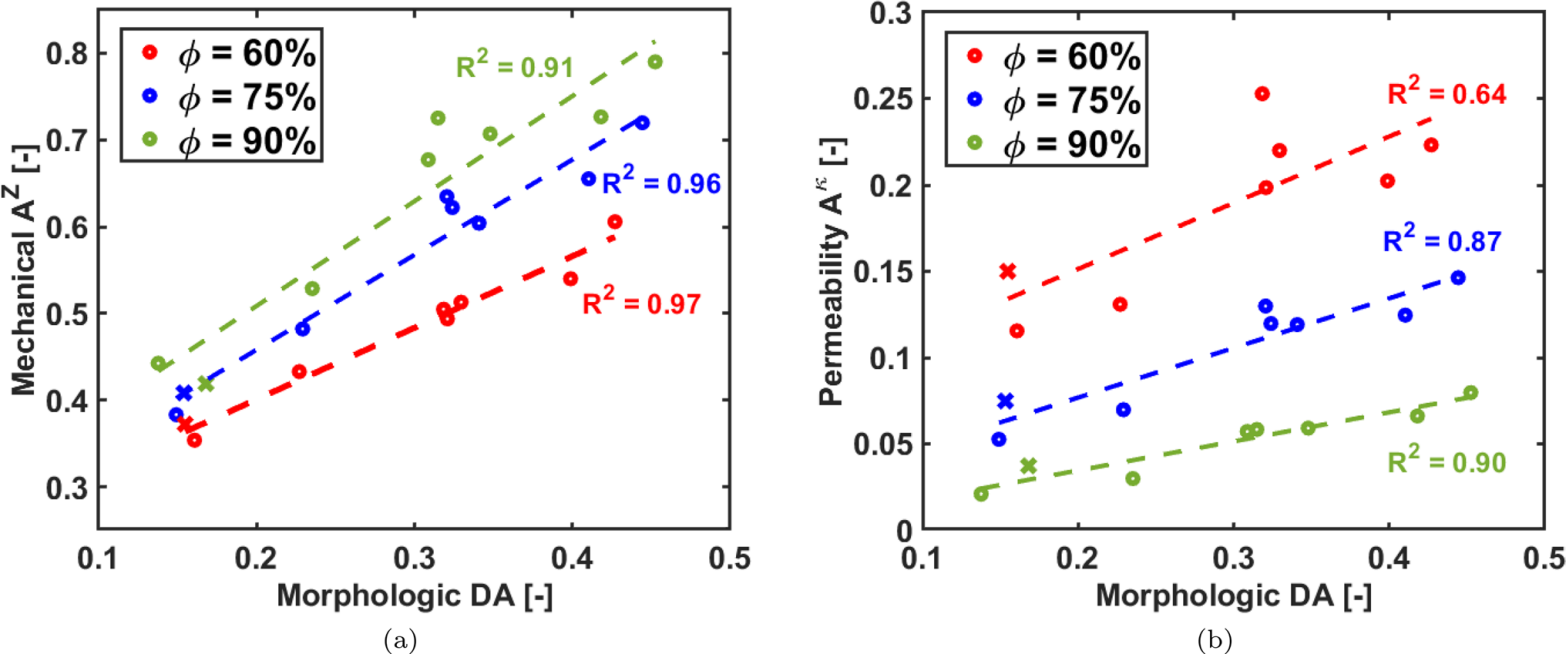
Panel **a**: correlation between *DA* and $A^Z$; panel **b**: correlation between *DA* and $A^\kappa$. The isotropic scaffolds are reported with the $\varvec{\times }$ symbol

The morphologic degree of anisotropy *DA* and the mechanical anisotropy $A^T$ correlate positively, as reported in Fig. 7a. The anisotropic permeability $A^\kappa$ also well correlates with the *DA* parameter (Fig. 7b). The correlation coefficient ($R^2$) for the $A^T$ parameter is almost constant among the three porosities, although a slight decrease is found as the porosity increases. As expected, the isotropic structures exhibit the lowest values of *DA* and $A^T$. In contrast to the *DA*-$A^T$ correlation, the $R^2$ for *DA*-$A^\kappa$ decreases as the porosity increases, this is consistent with the fact that macroscopic permeability is related with the pore space, instead of the solid component of the scaffold, which becomes predominant as the scaffold porosity increases.

### Fluid permeability

**Fig. 8:**
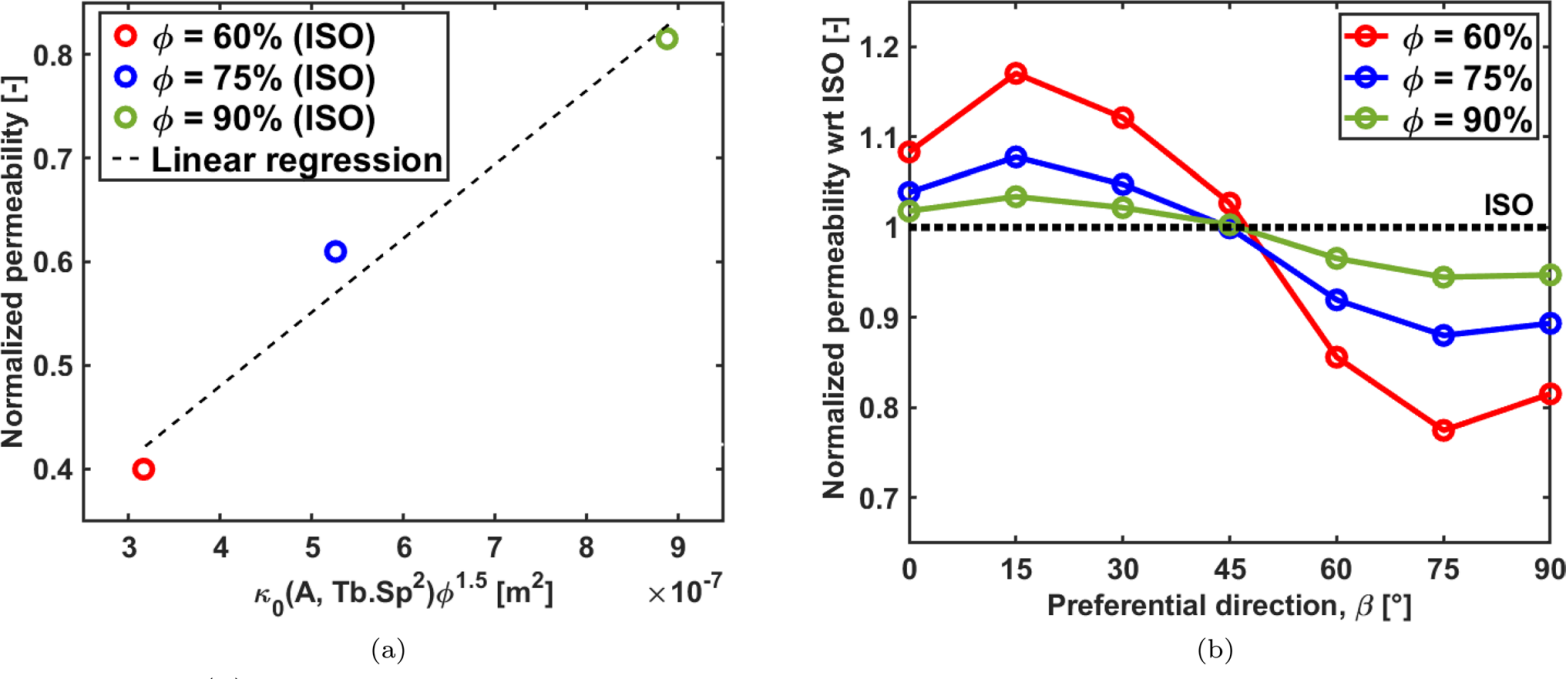
Panel **a**: correlation between the numerical permeability and the analytical model described in O’Brien et al. (2007). Data are obtained by setting $A=1$; panel **b**: comparison of the effective permeability of the isotropic scaffolds with respect to the permeability of the scaffolds exhibiting a preferential orientation of the trabeculae

In O’Brien et al. (2007) the following approximate relationship is found for isotropic macroscopic permeability (${\bar{\kappa }}$) of scaffolds with a micro-porous collagen foam in the pores, as a function of the porosity ($\phi$) and the pore average size (*d*):

26 $$\begin{aligned} {\bar{\kappa }}=Ad^2\phi ^{3/2} \end{aligned}$$

in which *A* is a dimensionless parameter which depends on the intrinsic parameter of the collagen foam ($\kappa _0$) and a characteristic length of the foam ($l_0$), as $A=\kappa _0/l_0^2$. The macroscopic permeability found through the finite element model on the isotropic scaffolds for the three porosities is consistent with the equation (26). Figure 8 (left panel) shows the dimensionless numerical permeability $\kappa _{ISO}^{FEM}/\kappa _0$ *vs* the functional variation defined by equation (26) $\frac{1}{\kappa _0}Ad^2\phi ^{3/2}=\frac{1}{l_0^2}Tb.Sp^2\phi ^{3/2}$. A linear relationship is found, thus confirming the same functional variation of the Voronoi scaffolds isotropic architecture with the morphometric parameters *Tb*.*Sp* and $\phi$.

Figure 8 (right panel) shows the macroscopic permeability along the vertical direction for all scaffolds with preferential orientation, normalized with respect to the permeability of the isotropic scaffold (no preferential orientation). As expected, scaffolds with trabeculae more aligned along the vertical directions ($\beta _z=0^{\circ },15^{\circ },30^{\circ }$) exhibit a permeability higher than that of the isotropic scaffold, up to a 20% increase for the less dense scaffold. Conversely, scaffolds with trabeculae more perpendicular to the vertical axis ($\beta _z=60^{\circ },75^{\circ },90^{\circ }$) exhibit a lower relative permeability with a minimum up to 20% reduction for the less dense scaffold. The scaffold $\beta _z=45^{\circ }$ exhibits a permeability which is approximately the same as that of the isotropic scaffold. The highest impact on trabecular orientation is found for the scaffolds with lowest density.

### Superficial octahedral strain

**Fig. 9:**
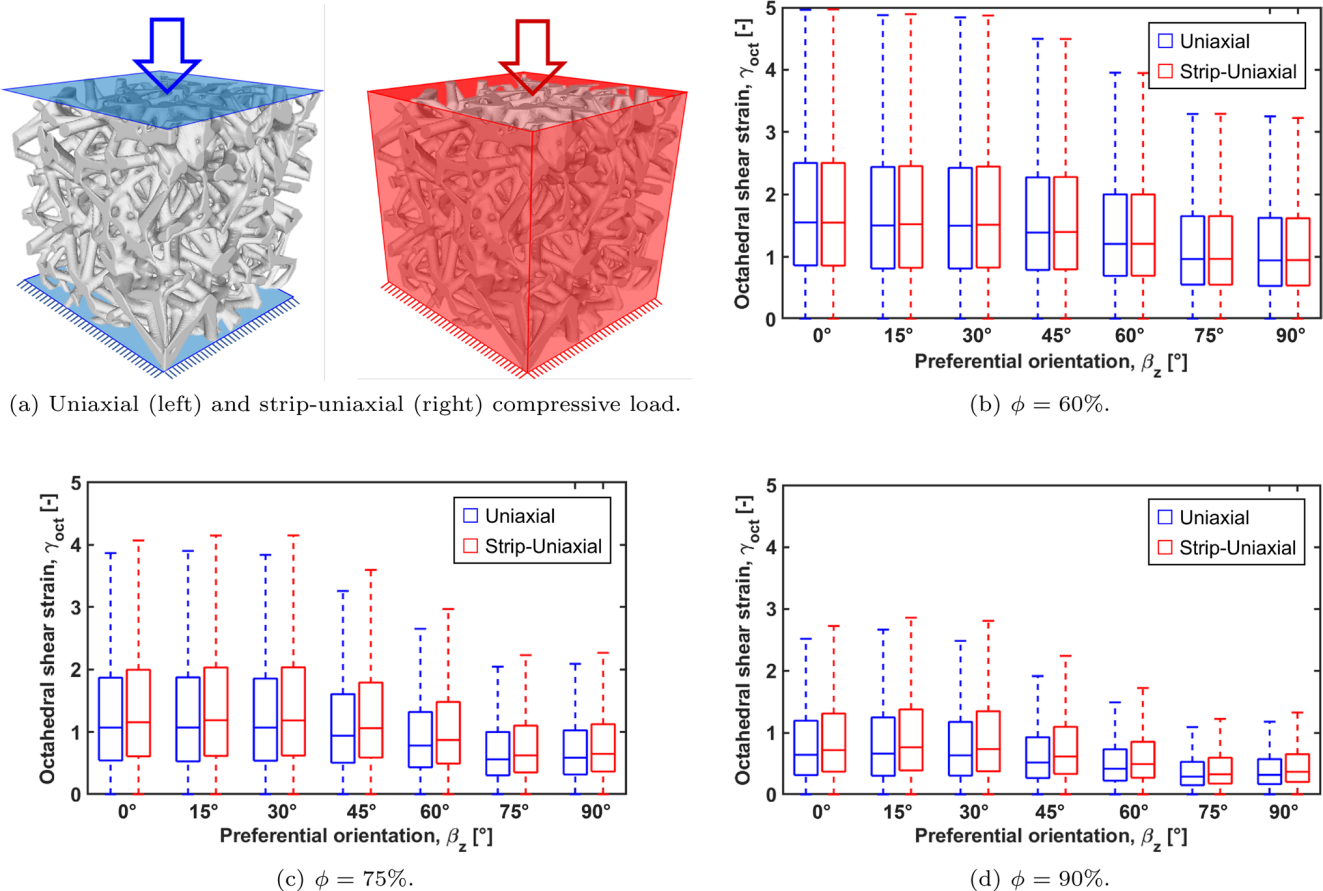
Box plot representing the median values of $\gamma _{oct}$ upon 1% macroscopic deformation (uniaxial and strip uniaxial). The three cases of porosity ($\phi$) are reported

The box plots of $\gamma _{oct}$ across the different preferential orientations upon a macroscopic compression of 1% (uniaxial load and strip uniaxial load) are reported in Fig. 9.

As expected, the $\gamma _{oct}$ depends on the preferential direction, as it influences the macroscopic stiffness of the scaffolds. In general, the lower the scaffold’s stiffness, the highest the $\gamma _{oct}$ in both the load configurations. The median value of $\gamma _{oct}$ is similar in both load configurations when the scaffolds with $\phi = 60\%$ are considered; when the porosity increases the strip uniaxial load exhibits higher median $\gamma _{oct}$, suggesting that the deformation mode of more porous scaffolds is more affected by external boundary conditions than denser structures.

## Discussion

The aim of this work was to provide an algorithm able to generate Voronoi-based scaffolds with a tunable stiffness anisotropy with independent control on trabecular thickness and porosity. This approach allows for the design of scaffolds that not only meet structural requirements that vary according to the anatomical location, but also take into account the morphology of the surrounding tissue. Furthermore, given that the device is not intended to serve as a permanent bone substitute, its morphological and mechanical characteristics should be carefully tuned to ensure optimal load-bearing capability and cellular proliferation in the short term. These properties are crucial during the initial healing phase, as they will eventually be replaced by the natural mechanical behavior of the regenerating bone once continuity is restored.

In this paper, a suitable procedure is implemented to create micro-architecture with selected preferential orientation of the trabeculae; while specified trabecular thickness and overall porosity was achieved by means of a constrained minimization of the surface energy. The constraint on the overall porosity and the target values of trabecular thickness were nicely achieved for all selected porosity values and preferential orientations. The scaffolds were characterized both in terms of stiffness anisotropy and in terms of potential capability to promote newly formed tissue in the pores by assessing overall fluid permeability and superficial strain. The porosity of the scaffolds was maintained in the range of 60 – 90%, which is often reported to be a beneficial interval for cells’ growth and proliferation (Jiao et al. 2023).

Figure 6(a) shows how the isotropic samples, for all the three values of porosity, lay on the curve generated by applying the Gibson and Ashby model for bending-dominated structures (Gibson and Ashby, 1982). As this latter takes into account the porosity only for the stiffness prediction, Fig. 6b shows how imposing a preferential trabecular orientation, significantly affects the stiffness values deviating from the Gibson and Ashby prediction.

Manufacturing processes impose limitations on the size of the architectural features like trabecular thickness which must be larger than the minimum printable features; the capability of the proposed method to design independently preferential material orientation, porosity and trabecular thickness as shown in Fig. 4 is of particular interest. In light of a multidisciplinary design process, where it is necessary to simultaneously optimize elastic properties and mechano-biology performance, all designed scaffolds have been described in terms of mechanical anisotropy, overall fluid permeability, geometric curvatures, and superficial strain distribution. Elastic properties of the scaffolds are crucial to mitigate stress shielding in the bone surrounding the scaffolds. Therefore a similarity with the host bone is sought in scaffold design.

In osteoporotic trabecular bone, tissue stiffness and degree of anisotropy are highly correlated (Bregoli et al. 2024). Conversely, the presented algorithm to generate Voronoi-based scaffold exhibits higher flexibility as independent design of stiffness and anisotropy can be achieved.

Vajjhala et al. (2000) performed a study, generating Voronoi structures through the Surface Evolver software (Brakke 1992). One of their major findings was that, removing trabeculae dramatically reduces the stiffness, whereas the thinning of the trabeculae has a less important effect. In our study, even if we remove trabeculae in the structure, the algorithm is able to select the trabeculae properly, such that the stiffness can increase with respect to the isotropic case.

Fantini and Curto (2018) run image analyses on Voronoi scaffolds, finding a degree of anisotropy equal to 0.05. Conversely, in our study, we found that the *DA* for the three classes of porosity is between 0.1 and 0.2, for scaffolds without any preferential orientation. A possible explanation of this discrepancy may be due to a much higher amount of trabeculae in the work of Fantini and Curto with respect to our study; indeed, a higher number of randomly oriented trabeculae corresponds to an isotropic configuration.

**Fig. 10:**
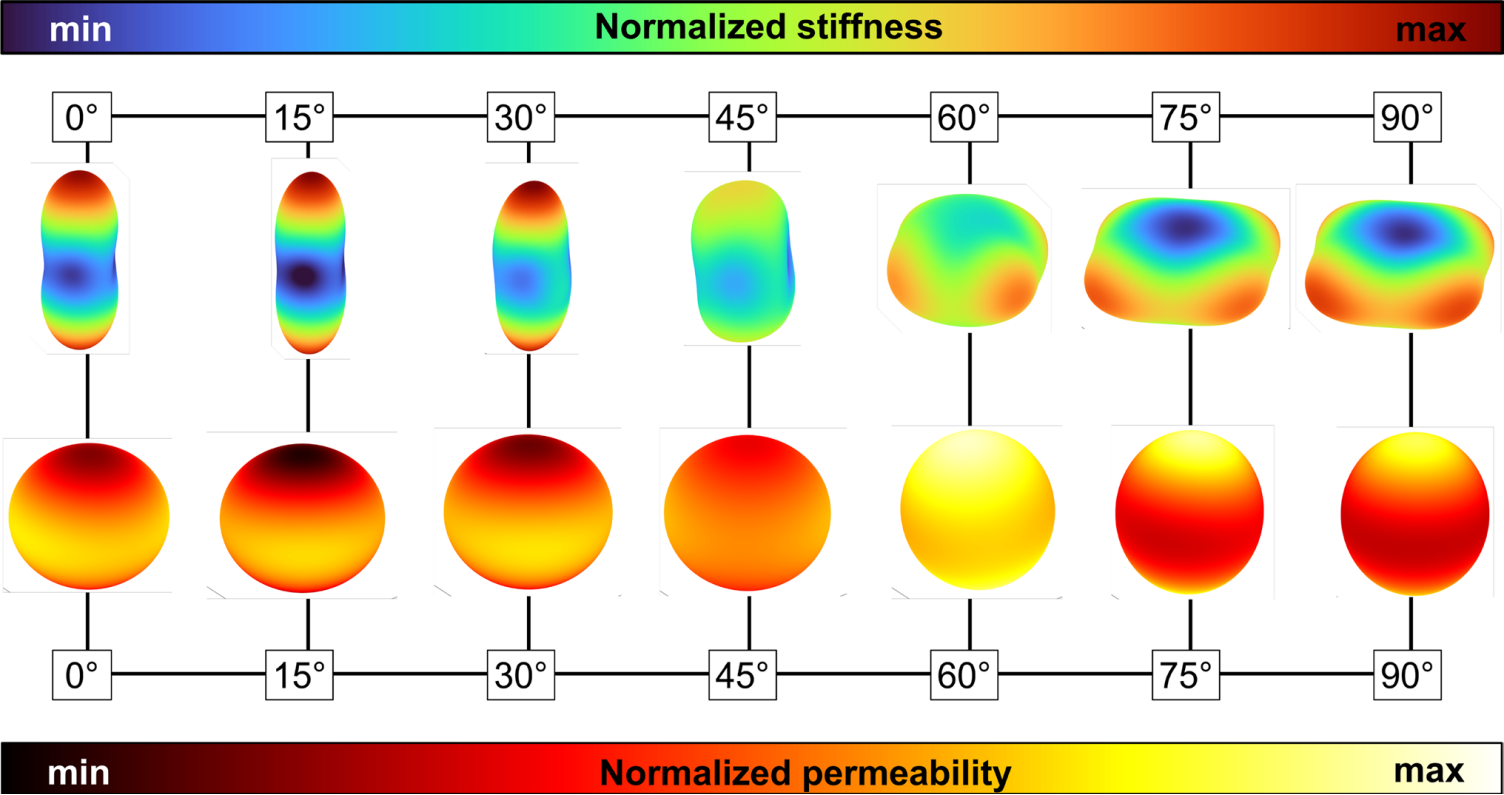
Three dimensional representation of the normalized homogenized stiffness and permeability for scaffolds exhibiting $\phi =75\%$

Considering the permeability trend reported in Fig. 8, it can be seen that the trabecular preferential orientation has a direct impact on the permeability. Since the trabecular orientation does not affect the *Tb*.*Sp*, the variation on the permeability is due to pore connectivity and shape of the pores. In Fig. 10, a comparison of the macroscopic stiffness and the macroscopic permeability in all spatial orientations and for all the scaffolds exhibiting a porosity of $75\%$ is presented.

Both permeability and elastic modulus are highly directional (high degree of anisotropy) for low preferential angle with respect to the z-axis. The z stiffness and permeability decreases with increasing preferential angle.

The $60^{\circ }$ configuration exhibits macroscopic properties more similar to the isotropic case, for both the quantities; considering the macroscopic stiffness, it assumes the lowest value of the $A^T$, and of $A^\kappa$. Since the porosity among different values of $\beta _z$ is constant, the variation of the macroscopic permeability is due to pore shape and pore orientation. On scaffolds with $\beta _z=75^{\circ }, 90^{\circ }$ elastic properties exhibit anisotropy also in the x-y plane; this anisotropy in the x-y plane is substantially weaker in the macroscopic fluid permeability. Although these two quantities play a role in the mechano-biology of bone cells, optimal values are still to be provided (Chauhan and Bhatt 2023).

Kong et al. (2024) investigated random architectures composed of rod-like and plate-like structural elements, demonstrating that plate-like features effectively reduce local stress concentrations. In this study, we did not include large plate-like structures because, although this feature is suitable for reducing the risk of mechanical failure of the scaffold, it may cause a reduction of the permeability.

Furthermore, while we do not directly control the quantity of plate-like structures, their occurrence is closely related to the porosity. Specifically, when trabeculae are in close proximity, they tend to merge, forming plate-like structures. Although in the presented Voronoi scaffold with few plate-like structures, the stress concentration can be generally unfavorable, the mechano-biological implications warrant further consideration. Maevskaia et al. (2023) implemented an algorithm that generates scaffolds with adaptive density minimal surfaces corresponding to plate-like structures with 3D curvature in space. While this approach is mechanically effective, the high tortuosity of the pores may result in an unfavorable permeability, affecting the cell proliferation in large bone defects. Controlling the placement of the plate-like structures in a Voronoi tessellation could be a good compromise between mechanically compatible scaffolds, optimizing in the meantime the cellular proliferation.

The latter aspect is highly dependent on the physical features of a scaffold. In particular, it has been observed that cells’ proliferation is stimulated when the scaffolds exhibit at least a negative principal curvature (Callens et al. 2023) or in other words, cells proliferate more in concave shapes, unlike convex or flat curvatures (Werner et al. 2016). In our work, we observed that all the scaffolds exhibit a dominant negative Gaussian curvature (i.e., one negative principal curvature, see Fig. 5). This trend is independent on the preferential orientation of the trabeculae (data not shown), thereby the preferential orientation can be selected during the design phase without impacting curvature, and, by extension, cellular response. This similarity arises primarily from the fact that the inter-trabecular angle remains constant irrespective of the orientation angle $\beta _z$, as well as the trabecular thickness. Consequently, there is no underlying reason for the curvature to vary based on scaffold orientation. Deep-learning techniques offer an additional tool for designing random structures by prescribing desired curvatures and generating mechanically compatible scaffolds (Guo et al. 2024). However, the strong dependency of these data-driven approaches on the training dataset highlights the necessity of a mathematical formulation to ensure control over anisotropic properties.

Before bringing this study to the in-vitro or in-vivo level, additional in-silico investigations need to be carried out addressing the key aspects that were not investigated in this work. One of them is represented by the mechanical failure of the scaffolds; indeed, in a similar trabecular-like scaffolds, D’Andrea et al. (2023) found that *Tb*.*Th*, the ellipsoid factor and the orientation of slender trabeculae play a role in the fracture behavior of the structure. Since in this work the *Tb*.*Th* is constant among the preferential directions, it can be speculated that structures exhibiting a $\beta _z < 20^{\circ }$ may be stronger than the other orientation. However, a suitable study investigating the strength of the scaffold should be performed. The second aspect is represented by a direct evaluation of the cellular proliferation and tissue differentiation. Different approaches can be applied for the predictions of the cellular proliferation: (i) by using mathematical models to relate the pores dimensions and the stiffness of the scaffolds to the bone ingrowth in a dynamic environment (Annunziata et al. 2023), (ii) phenomenological models based on the assumption that the cellular proliferation is affected mainly by the stiffness of the scaffold (Nasello et al. 2021), and (iii) explicitly simulating individual bone cells and their interaction with each other and the effect of the surrounding environment on the bone growth (Faweya et al. 2022).

While this study presents significant insights derived from numerical simulations, it is critical to recognize the lack of experimental verification as a central limitation. To address this, precise delineation of the experimental framework is essential, as it is imperative to conduct tests on realistically micro-structured samples accounting for the peculiarities of the constituent material. For example, evaluating the elastic response of such micro-structured ceramics poses substantial challenges, rendering conventional loading methods like uniaxial compression inadequate in terms of reliability. Therefore, alternative methodologies, such as acoustic techniques, should be explored (see for example D’Andrea et al. 2025). Furthermore, permeability assessments and cell culture trials are anticipated to be scheduled in subsequent phases of the research.

## Conclusion

This work focused on the generation of Voronoi architectures for bone tissue engineering. The design algorithm introduces the possibility to control the preferential trabecular orientation inside the scaffold which allows to tune the scaffold’s geometry and mechanical behavior. The correct functioning of the algorithm allowed for the decoupling of the mechanical properties with respect to the porosity, that are typically closely connected. In particular, the macroscopic stiffness, macroscopic permeability and octahedral shear strain are the only three parameters that are affected by the preferential orientation of the trabeculae, whereas, the trabecular thickness, trabecular spacing, porosity and curvatures are independent of the trabecular orientation. Even if we designed and analyzed three values of porosity, the robustness of the design algorithm is able to provide any value of porosity. Although the result’s normalized form permits a broad application of the findings, specific consideration will be required based on the constituent material. Finally, future work incorporating experimental mechanical testing and cell culture will strengthen the findings of this paper.

## Data Availability

The developed MATLAB codes to generate Voronoi scaffolds are available at https://zenodo.org/records/15037418.
